# Liver Cell Mitophagy in Metabolic Dysfunction-Associated Steatotic Liver Disease and Liver Fibrosis

**DOI:** 10.3390/antiox13060729

**Published:** 2024-06-15

**Authors:** Jiaxin Chen, Linge Jian, Yangkun Guo, Chengwei Tang, Zhiyin Huang, Jinhang Gao

**Affiliations:** 1 Laboratory of Gastroenterology and Hepatology, West China Hospital, Sichuan University, Chengdu 610041, Chinatangcw@scu.edu.cn (C.T.); 2 Department of Gastroenterology, West China Hospital, Sichuan University, Chengdu 610041, China

**Keywords:** metabolic dysfunction-associated steatotic liver disease (MASLD), metabolic dysfunction-associated steatohepatitis (MASH), liver fibrosis, mitochondria, mitophagy, hepatocytes, hepatic stellate cells (HSCs), Kupffer cells (KCs), macrophages, liver sinusoidal endothelial cells (LSECs)

## Abstract

Metabolic dysfunction-associated steatotic liver disease (MASLD) affects approximately one-third of the global population. MASLD and its advanced-stage liver fibrosis and cirrhosis are the leading causes of liver failure and liver-related death worldwide. Mitochondria are crucial organelles in liver cells for energy generation and the oxidative metabolism of fatty acids and carbohydrates. Recently, mitochondrial dysfunction in liver cells has been shown to play a vital role in the pathogenesis of MASLD and liver fibrosis. Mitophagy, a selective form of autophagy, removes and recycles impaired mitochondria. Although significant advances have been made in understanding mitophagy in liver diseases, adequate summaries concerning the contribution of liver cell mitophagy to MASLD and liver fibrosis are lacking. This review will clarify the mechanism of liver cell mitophagy in the development of MASLD and liver fibrosis, including in hepatocytes, macrophages, hepatic stellate cells, and liver sinusoidal endothelial cells. In addition, therapeutic strategies or compounds related to hepatic mitophagy are also summarized. In conclusion, mitophagy-related therapeutic strategies or compounds might be translational for the clinical treatment of MASLD and liver fibrosis.

## 1. Introduction

Metabolic dysfunction-associated steatotic liver disease (MASLD), formerly known as non-alcoholic fatty liver disease [1], is the most common liver disease and the leading cause of liver-related morbidity worldwide [2]. Approximately one-third of the population suffers from MASLD, and the incidence of MASLD is increasing in parallel with the growth of metabolic syndrome, including obesity and type 2 diabetes [3]. MASLD is widely recognized as a multisystem disease with potential hepatic and extrahepatic outcomes, including cardiovascular, metabolic, oncological, and other complications [4]. The hepatic manifestation of MASLD is characterized by an accumulation of fat and inflammation, encompassing disease states from simple lipid accumulation or steatosis (metabolic dysfunction-associated steatotic liver, MASL) to its active inflammatory form, metabolic dysfunction-associated steatohepatitis (MASH) [5,6]. The persistence of MASH even further progresses to liver fibrosis, cirrhosis, and hepatocellular carcinoma (HCC) in some cases [5]. Despite considerable investigations into the underlying mechanisms, only resmetirom is now approved by the U.S. Food and Drug Administration (FDA) for the clinical treatment of MASH [7]. Therefore, further studies that focus on the mechanism of MASLD to find new drugable targets, and adequate summaries are needed.

The liver is a crucial organ for macronutrient and drug metabolism, immune homeostasis, and endocrine regulation. These complex physiological processes are mainly performed by hepatocytes, hepatic stellate cells (HSCs), Kupffer cells (KCs)/macrophages, and liver sinusoidal endothelial cells (LSECs). Mitochondria are highly dynamic organelles essential for fatty acid oxidation, lipogenesis, and gluconeogenesis in these liver cells, ranging from substrate metabolism to energy production [8]. Thus, disturbances in mitochondrial homeostasis, such as altered mitochondrial morphology and function, altered mitochondrial quality control, and mitophagy, have been demonstrated to be involved in the pathogenesis of MASLD and liver fibrosis [8,9,10]. Mitophagy, a selective form of autophagy, enables the removal and recycling of dysfunctional or superfluous mitochondria and modulates mitochondrial biogenesis [11]. Mitophagy favors the routine β-oxidation of lipids in healthy mitochondria, while impaired mitophagy can lead to more severe metabolic disorders, such as obesity, insulin resistance, and MASLD [12,13].

Although significant advances in mitophagy in liver diseases have been summarized in several recently published reviews [14,15,16,17,18], only one mini-review has focused on mitophagy in MASLD [18]. Additionally, mitophagy in different types of liver cells influences the progression of MASLD and liver fibrosis. However, the contribution of mitophagy in specific liver cells to the pathogenesis of MASLD and liver fibrosis has not been summarized. In this review, we comprehensively summarize the role of liver cell mitophagy, including that of hepatocytes, HSCs, KCs/macrophages, and LSECs, in the development of MASLD and liver fibrosis. The promising strategies or compounds targeting mitophagy are also summarized. By organizing the research in a liver cell mitophagy-based structure, we will comprehensively explore the role and mechanism of liver cell mitophagy in MASLD and liver fibrosis.

## 2. Mitophagy

### 2.1. Concept of Mitophagy

Autophagy is a lysosome-dependent catabolic process. Autophagy is crucial for maintaining cellular homeostasis through the digestion and recycling of intracellular components, including impaired organelles and aberrantly accumulated materials such as aggregated proteins and lipid droplets [19]. Autophagy can function in nonselective or selective ways. Nonselective autophagy randomly engulfs cytoplasmic components into autophagosomes [20], whereas selective autophagy degrades substances recognized by autophagy receptors [21]. Mitophagy, a subtype of selective autophagy that removes damaged mitochondria, was first described by John Lemasters in 2005 [22]. Functionally, it is generally accepted that mitophagy is responsible for the selective removal and recycling of impaired mitochondrial components as well as the biogenesis of new fully functional mitochondria [11,21].

### 2.2. Processes of Mitophagy

**Figure 1:**
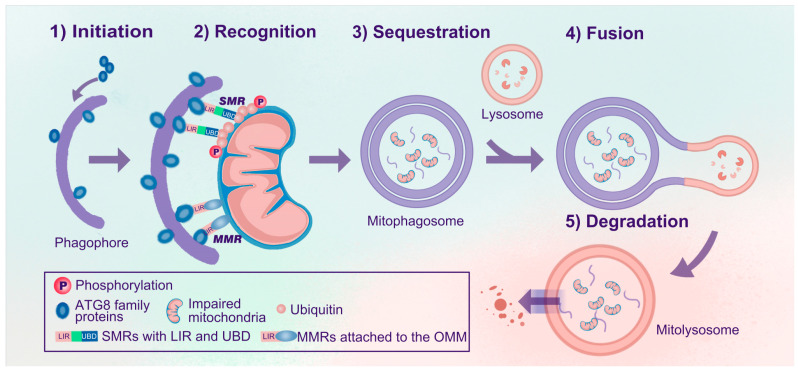
Processes of mitophagy. The process of mitophagy can be divided into five steps: (1) Initiation: ATG8 family proteins are anchored in the inner and outer membranes of the phagophores. (2) Recognition: SMRs or MMRs recognize impaired mitochondria and link the mitochondria to phagophores via the ATG8-LIR interaction. (3) Sequestration: Impaired mitochondria are sequestered, forming mitophagosomes. (4) Fusion: Mitophagosomes further fuse with lysosomes to form mitolysosomes. (5) Degradation: Impaired mitochondria are degraded and recycled in mitolysosomes. Abbreviations: Atg, autophagy-related gene; SMRs, soluble mitophagy receptors; MMRs, membrane-attached mitophagy receptors; UBDs, ubiquitin-binding domains; LIR, microtubule-associated protein 1A/1B light chain 3 (LC3)-interacting region (LIR); OMM, outer mitochondrial membrane.

Mitophagy selectively degrades dysfunctional or superfluous mitochondria. Generally, mitophagy can be divided into five steps: (1) initiation, (2) recognition, (3) sequestration, (4) fusion, and (5) degradation (Figure 1). In the initiation step, ATG8 homologs (LC3/GABARAPs) form covalent bonds with phosphatidylethanolamine enriched in the phagophores, thus forming lipidated ATG8 (e.g., LC3-II) and anchoring them to the inner and outer membranes of the phagophores. Efficient recognition is essential for mitophagy, similar to other types of selective autophagy [23]. During autophagy, soluble or membrane-attached cargo receptors recognize cargo. Then, cargo-bound receptors interact with lipidated ATG8 family proteins anchored in the membrane at the concave side of the phagophores [19]. Accordingly, mitophagy receptors can be classified into soluble mitophagy receptors (SMRs) and membrane-attached mitophagy receptors (MMRs) (Figure 1) [14]. SMRs can bind to ubiquitylated outer mitochondrial membrane (OMM) proteins and are often related to ubiquitin-dependent mitophagy, whereas MMRs are localized to the mitochondrial membrane and are ubiquitin-independent. SMRs have specific ubiquitin-binding domains (UBDs) and microtubule-associated protein light chain 3 (LC3)-interacting region (LIR) motifs, while most MMRs have integral LIR motifs but no UBDs [14,19,24]. SMRs and MMRs are tightly linked to mitochondria and anchored to phagophores via interactions between LIR motifs and lipidated ATG8 family proteins [14,19,21].

Subsequently, mitochondria are sequestered, forming mitophagosomes. These mitophagosomes are similar to autophagosomes, but the former are specific for sequestering mitochondria, whereas autophagosomes engulf a broader range of cargo. Eventually, mitophagosomes fuse with acidic lysosomal compartments to degrade and recycle mitochondria in mitolysosomes.

### 2.3. Signaling Pathway of Mitophagy

Considering the dependence of mitophagy on ubiquitin and different mitophagy receptors, mitophagy signaling pathways are classified into PINK1/Parkin-dependent, PINK1/Parkin-independent, and other mitophagy signaling pathways.

(1) PINK1/Parkin-dependent mitophagy signaling pathway

**Figure 2:**
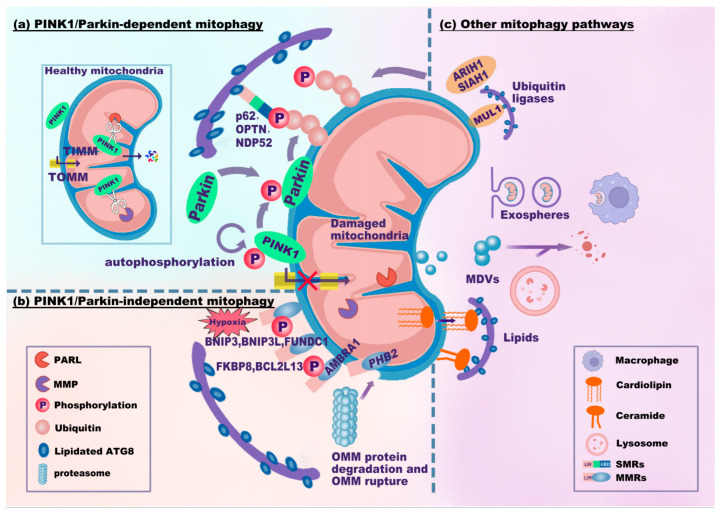
Mitophagy signaling pathways. The mitophagy signaling pathways can be classified as PINK1/Parkin-dependent (**a**), PINK1/Parkin-independent (**b**), or other mitophagy signaling pathways (**c**). (**a**) In healthy mitochondria, PINK1 translocates into mitochondria through TOMM and TIMM and is proteolytically cleaved by PARL and MPP. Upon mitochondrial damage, PINK1 fails to enter and accumulates on the OMM, which recruits and phosphorylates Parkin. PINK1 and Parkin jointly generate p-Ub chains on the OMM. SMRs (e.g., p62, OPTN, and NDP52) recognize p-Ub signals and link damaged mitochondria to phagophores via the LIR-ATG8 interaction. (**b**) MMRs directly interact with lipidated ATG8. BNIP3, BNIP3L, and FUNDC1 are common in response to hypoxia. PHB2 localizes to the IMM. Upon proteasome-mediated rupture of the OMM, PHB2 interacts with ATG8. (**c**) Several ubiquitin ligases, MUL1, SIAH1, and ARIH1, ubiquitinate the OMM to promote mitophagy. Notably, MUL1 has an LIR motif that interacts with ATG8. Damaged mitochondria can be translocated to other cells (e.g., macrophages) through exospheres or EVs for mitophagy. MDVs derived from mitochondria fuse with lysosomes for degradation. Cardiolipin translocates from the IMM to the OMM to interact with ATG8. Abbreviations: TOMM/TIMM, translocase complex of the outer/inner mitochondrial membrane (OMM/IMM); PARL, presenilin-associated rhomboid-like protein; MPP, mitochondrial processing peptidase; p-Ub, phosphorylated ubiquitin; MDVs, mitochondria-derived vesicles; EVs, extracellular vesicles.

In mitophagy, the phosphatase and tensin homolog (PTEN)-induced kinase (PINK)1/Parkin-dependent signaling pathway is the best characterized and canonical signaling pathway (Figure 2a). PINK1 is a mitochondrial serine/threonine kinase, while Parkin is an E3 ubiquitin (Ub) ligase. In healthy mitochondria with normal membrane potential, PINK1 translocates into mitochondria through the translocase complex on the OMM (TOMM) and the inner mitochondrial membrane (TIMM). Then, PINK1 is proteolytically cleaved and inactivated by presenilin-associated rhomboid-like protein (PARL) and mitochondrial processing peptidase (MPP) [25,26]. The cleavage product of PINK1 translocates to the cytosol and inhibits Parkin translocation to the mitochondria [27]. Upon mitochondrial injury, dysfunctional mitochondria (failure of mitochondria to function normally) exhibit a depolarized mitochondrial membrane potential (MMP). Depolarized mitochondria result in the selective accumulation of PINK1 at the OMM [28,29], and PINK1 is essential for recruiting Parkin [28]. Parkin (encoded by the gene *Park2*) translocates to depolarized mitochondria and mediates the subsequent engulfment and elimination of dysfunctional mitochondria [30]. In addition, PINK1 accumulation leads to the phosphorylation of Parkin, ubiquitin, and OMM proteins [29,31,32,33,34]. It seems that PINK1 can activate Parkin through recruitment and phosphorylation. Activated Parkin promotes the ubiquitination of OMM proteins and builds ubiquitin chains [32]. OMM proteins and Parkin-generated Ub tags can be degraded by proteasome-dependent pathways, leading to the rupture of the OMM [35,36]. In addition, these ubiquitin chains are phosphorylated by PINK1, resulting in the formation of phosphorylated ubiquitin (p-Ub) chains. The p-Ub chains can further activate Parkin, forming a Parkin-p-Ub positive feedback loop [34]. SMRs can recognize all these p-Ub signals, eventually leading to mitophagy.

The key ubiquitin-dependent SMRs include p62/SQSTM1/sequestosome-1, calcium-binding and coiled-coil domain 2 (CALCOCO2, also known as NDP52), and optineurin (OPTN). These SMRs recognize p-Ub signals and target mitochondria to phagophores via the LIR-ATG8 interaction. The autophagic adaptor p62 is indispensable for final clearance in PINK1/Parkin-dependent mitophagy [37]. Parkin-dependent or Parkin-independent p-Ub can recruit NDP52 and OPTN. Moreover, NDP52 and OPTN attract autophagy factors upstream of LC3 close to mitochondria, including UNC-51-like kinase 1 (ULK1), double FYVE-containing protein 1 (DFCP1), and WD repeat domain phosphoinositide-interacting protein 1 (WIPI1) [32]. OPTN can also form a complex with ATG9a vesicles to initiate PINK1/Parkin-dependent mitophagy [38]. Additionally, LC3/GABARAPs can mediate the ubiquitin-independent recruitment of OPTN and NDP52 to amplify mitophagy [39]. These results indicate that NDP52 and OPTN are crucial for the mitophagy machinery.

Overall, the PINK1/Parkin-dependent mitophagy signaling pathway involves the stabilization of PINK1 on the OMM and subsequent recruitment of Parkin. PINK1 and Parkin jointly contribute to the generation of p-Ub chains on OMM proteins. SMRs (p62, OPTN, and NDP52) recognize p-Ub signals and link mitochondria to phagophores via the LIR-ATG8 interaction, eventually leading to mitophagy.

(2) PINK1/Parkin-independent mitophagy signaling pathway

The PINK1/Parkin-independent mitophagy signaling pathway mainly involves ubiquitin-independent MMRs attached to the mitochondrial membrane. Most of the MMRs have integral LIR motifs. These LIR motifs can bind to lipidated ATG8 family proteins at the phagophore membrane, bypassing the need for ubiquitination (Figure 2b). MMRs primarily include BCL2 interacting protein 3 (BNIP3), BNIP3-like protein (BNIP3L/NIX), BCL2-like protein 13 (BCL2L13), FUN14 domain containing 1 (FUNDC1), FKBP prolyl isomerase 8 (FKBP8), and prohibitin 2 (PHB2) [24].

BNIP3, BNIP3L/NIX, and FUNDC1 are localized to the OMM and are commonly expressed in response to various stimuli (e.g., hypoxia) [40,41]. BNIP3 and BNIP3L contain a BCL2 homology (BH) domain 3 and are thus identified as pro-apoptotic proteins [42]. BNIP3 can stabilize PINK1 on the OMM by suppressing its proteolytic cleavage [43]. BNIP3L clears damaged mitochondria through its amino-terminal LIR motif, which recruits and interacts with GABARAP-L1 [44]. Once phosphorylated at serine residues (Ser17 and Ser24), the LIR of BNIP3L further enhances its interaction with LC3/GABARAP proteins and promotes mitophagy [45,46]. Interestingly, both BNIP3 and BNIP3L can enhance the recruitment of Parkin [47,48]. BNIP3L even acts as a substrate of Parkin, and ubiquitinated BNIP3L further recruits NBR1 to promote mitophagy [49]. Thus, there is a crosstalk between ubiquitin-independent mitophagy receptors (BNIP3 and BNIP3L) and PINK1/Parkin-dependent signaling pathways. FUNDC1, whose typical LIR (18–YxxL–21) motif interacts with LC3B, is known to be a receptor for hypoxia-induced mitophagy [41]. ULK1 translocates to damaged mitochondria and phosphorylates FUNDC1 at serine 17 to enhance the LIR-LC3 interaction [50]. However, dephosphorylation at specific sites is also essential for FUNDC1-induced mitophagy. Phosphoglycerate mutase 5 (PGAM5), a phosphatase localized in mitochondria, dephosphorylates FUNDC1 at serine 13 to enhance the LIR-LC3 interaction upon hypoxia or carbonyl cyanide 4-(trifluoromethoxy)-phenylhydrazone (FCCP) treatment [51]. The phosphorylation status of specific FUNDC1 sites modulates the LIR-LC3 interaction, thus affecting FUNDC1-mediated mitophagy [52,53].

Other MMRs, such as BCL2L13, FKBP8, and PHB2, can also induce mitophagy. As a mammalian Atg32 homolog, BCL2L13 has two LIR motifs and was shown to mediate mitophagy and mitochondrial fragmentation [54,55]. FKBP8 belongs to the FK506-binding protein (FKBP) family. Although FKBP8 has no phosphorylatable residue in its LIR motif, FKBP8 can recruit LC3A and mediate mitophagy [24,56]. PHB2 is a mitophagy receptor localized to the IMM. Once mitochondria are impaired or depolarized, PHB2 can bind to the PARL protease to prevent proteolytic cleavage of PGAM5. Then, PGAM5 helps stabilize PINK1 at the OMM, followed by activation of the PINK1/Parkin-dependent mitophagy signaling pathway and proteasome-mediated rupture of the OMM. The exposed PHB2 binds to LC3B in phagophores via the LIR-LC3B interaction [57,58]. Strictly speaking, PHB2 is a PINK1/Pakin-dependent MMR.

In addition, autophagy/beclin 1 regulator 1 (AMBRA1) may also contribute to mitophagy via PINK1/Parkin-dependent or -independent signaling pathways. AMBRA1 translocates to perinuclear clusters of depolarized mitochondria and activates the class III phosphatidylinositol 3-kinase (PI3K) complex, facilitating the formation of phagophores [59]. AMBRA1 can localize to the OMM and bind to LC3 through the LIR motif [60]. Thus, AMBRA1 either promotes Parkin-dependent mitophagy by interacting with Parkin [59] or induces PINK1/Parkin-independent mitophagy via direct LIR-LC3 interaction [60]. Phosphorylated AMBRA1, induced by the E3-ubiquitin ligase HUWE1 (HECT, UBA, and WWE domain containing E3 ubiquitin protein ligase 1)-kappa B kinase (IKK) α, is fundamental for the LIR-LC3 interaction [61].

Generally, the PINK1/Parkin-independent mitophagy signaling pathway mainly depends on MMRs (BNIP3, BNIP3L/NIX, BCL2L13, FUNDC1, FKBP8, and PHB2). MMRs can directly bind to lipidated ATG8 family proteins through their LIR motifs. However, MMRs, such as BNIP3, BNIP3L, and PHB2, are not entirely independent of PINK1 or Parkin. The coordination between MMRs and the PINK1/Parkin-dependent mitophagy signaling pathway is worthy of further investigation.

(3) Other mitophagy signaling pathways

In addition to the canonical MMR-mediated PINK1/Parkin-independent mitophagy signaling pathway, several ubiquitin ligases and lipids related to mitochondrial clearance can also regulate mitophagy in a Parkin-independent manner (Figure 2c). These mitophagy-related ubiquitin ligases include mitochondrial E3 ubiquitin protein ligase 1 (MUL1), Siah E3 ubiquitin protein ligase 1 (SIAH1), and Ariadne RBR E3 ubiquitin protein ligase 1 (ARIH1). MUL1 is embedded in the OMM with its RING finger domain (containing the LIR motif) floating in the cytoplasm. MUL1 can enhance mitophagy either by ubiquitinating OMM or interacting with GABARAP [62]. In addition, MUL1 mediates selenite-induced mitophagy by promoting the ubiquitination of ULK1 [63]. SIAH1 is involved in the PINK1-synphilin-1-SIAH1 mitophagy pathway independent of Parkin. The PINK1-synphilin-1 complex is recruited to mitochondria, and synphilin-1 enhances the accumulation of PINK1 by further depolarizing mitochondria [64]. Moreover, synphilin-1 recruits SIAH1 to ubiquitinate mitochondrial proteins and to facilitate subsequent mitophagy [64]. ARIH1 was shown to trigger mitophagy in cancer cells by polyubiquitinating impaired mitochondria. This ARIH1-mediated mitophagy protects cancer cells against chemotherapy-induced cytotoxicity [65].

Lipids have also been shown to mediate mitophagy. Ceramide-induced mitophagy is mediated by dynamin-related protein 1 (Drp1)-dependent mitochondrial fission and involves direct interaction between ceramide and LC3 β-lipidation II (LC3B-II) [66]. Cardiolipin, an inner mitochondrial membrane phospholipid, acts as an ‘eat-me’ signal of mitophagy for clearance of dysfunctional mitochondria through translocating from the IMM to the OMM and interacting with LC3 [67]. Both ceramide and cardiolipin can recruit the autophagic machinery to dysfunctional mitochondria, eventually enhancing mitophagy. In addition to lipids, a mitophagy signaling pathway dependent on LC3C was also identified. Selected proteins in mitochondria, such as metaxin 1 (MTX1, located in the OMM), are targeted by LC3C and p62 through a piecemeal mitophagy pathway [68].

Furthermore, exospheres, extracellular vesicles (EVs), or mitochondria-derived vesicles (MDVs) may also be involved in mitophagy. Dysfunctional mitochondria can be ejected from cells via exospheres or large EVs and are ultimately engulfed and degraded via mitophagy in macrophages [69,70]. The biogenesis of MDVs, which depends on PINK1/Parkin, enables cargo delivery from mitochondria to lysosomes [71,72,73]. This trafficking mechanism may help clear impaired mitochondria with normal membrane potential.

In summary, mitophagy is induced upon mitochondrial damage through several types of signaling pathways, including the PINK1/Parkin-dependent or PINK1/Parkin-independent pathways and other mitophagy signaling pathways. As the classification standards vary and there is close crosstalk among different signaling pathways, they cannot be distinguished entirely. Certain signaling pathways are induced in response to specific stimuli. For example, mitophagy related to MMRs (BNIP3, BNIP3L/NIX, and FUNDC1) is common under hypoxic conditions. Although hypoxia-induced damaged mitochondria are highly ubiquitinated, PINK1/Parkin and five ubiquitin-binding SMRs are dispensable for oxidation-induced mitophagy [74]. Due to the complexity of mitophagy signaling pathways, further analyses and adequate summaries are needed to understand mitophagy in certain cells and diseases.

## 3. Role of Liver Cell Mitophagy in MASLD and Liver Fibrosis

Mitochondrial dysfunction plays a significant role in the progression of MASLD, from simple lipid accumulation or steatosis to its active inflammatory form, fibrosis/cirrhosis, and HCC [8,9,10]. In response to mitochondrial dysfunction, selective mitochondrial autophagy occurs to remove damaged mitochondria [10]. However, emerging evidence suggests that impaired mitophagy is a feature of MASLD and liver fibrosis [14,15,16,17,18,75]. Since there are various cell types in the liver, mitophagy in different liver cells may have distinct effects on the progression of MASLD and liver fibrosis. Herein, we aim to provide an overview of mitophagy in different liver cells in MASLD and liver fibrosis.

### 3.1. Hepatocyte Mitophagy in MASLD

**Figure 3:**
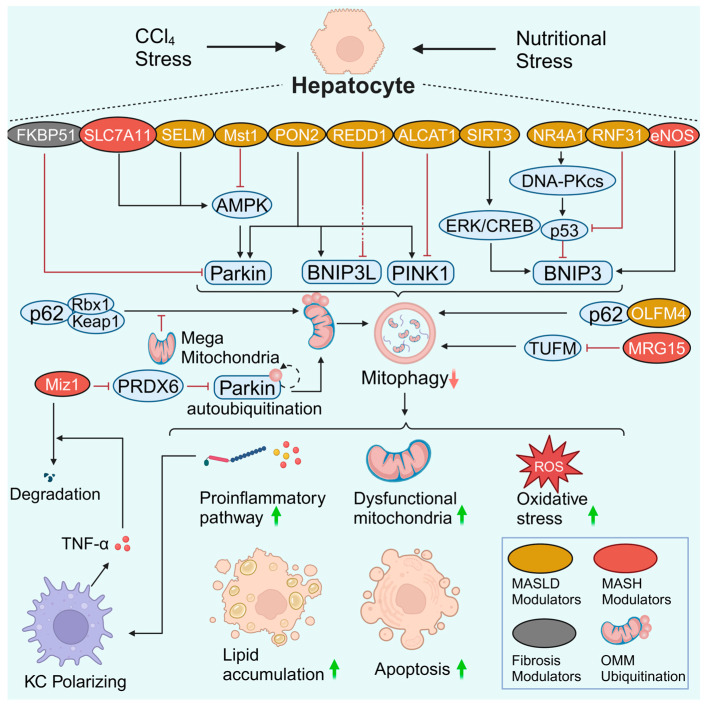
Hepatocyte mitophagy in MASLD and liver fibrosis. Many regulators and signaling pathways are involved in hepatocyte mitophagy in MASLD and liver fibrosis. In most cases, damaged hepatocyte mitophagy leads to the aggravation of inflammation, the accumulation of dysfunctional mitochondria, enhanced oxidative stress, lipid accumulation, and apoptosis. Notably, there is a crosstalk between hepatocytes and macrophages. A reduction in Miz1 results in increased levels of free PRDX6. PRDX6 interacts with Parkin and blocks Parkin autoubiquitination as well as downstream OMM ubiquitination. Impaired mitophagy caused hepatocyte inflammasome activation and stimulated macrophage TNF-α production. TNF-α further promotes Miz1 degradation in hepatocytes, forming a vicious feedback loop. Created with BioRender.com (accessed on 29 May 2024).

Hepatocytes are the central metabolic cells in the liver. Normal hepatic mitochondria are essential for energy production via the oxidation of glucose, lipids, and proteins. However, aberrant mitochondrial function contributes to impaired fatty acid oxidation and enhanced oxidative stress in hepatocytes [8]. Notably, the enhanced oxidative stress triggered by dysfunctional mitochondria and impaired mitophagy causes injury and even mitochondria-dependent apoptosis in hepatocytes [76,77]. Hepatocyte injury or death leads to inflammation of KCs or infiltrating macrophages and subsequent activation of HSCs, which favors the progression of MALSD [78]. An adequate summary of hepatocyte mitophagy is highly important (Figure 3). This section discusses the role of hepatocyte mitophagy and related signaling pathways in the pathogenesis of MASLD. The related mitophagy signaling pathways include the AMPK, PRDX6, p62, BNIP3, and other signaling pathways. Lipophagy is another type of selective autophagy that plays a prominent role in MASLD pathogenesis. The interplay between mitophagy and lipophagy is also briefly discussed.

***AMPK signaling pathway***

AMPK serves as a hub molecule for signal crosstalk to control mitochondrial health. Robust data confirmed that the AMPK signaling pathway modulates mitophagy in MASLD. As a member of the amino acid transporter family, solute carrier family 7 member 11 (SLC7A11) is a protective factor that is up-regulated in patients and mice with MASH. SLC7A11 decreases reactive oxygen species (ROS) levels and increases α-ketoglutarate (αKG)/proly hydroxylase (PHD) activity, thereby activating the AMPK-mitophagy axis and suppressing the expression of NLRP3 inflammation components. The SLC7A11-ROS/αKG-AMPK axis in hepatocytes is pivotal for controlling MASH progression, whereas *Slc7a11* knockdown or knockout aggravates steatohepatitis [79]. Macrophage stimulating 1 (Mst1) is an upstream regulator of mitophagy that decreases Parkin expression via repression of the AMPK signaling pathway. In contrast, genetic ablation of *Mst1* activates the AMPK pathway and reverses Parkin-related mitophagy, eventually attenuating high-fat diet (HFD)-mediated mitochondrial stress and hepatocyte apoptosis [77]. However, whether Mst1 can regulate AMPK-related mitophagy via other mitophagy signaling pathways needs to be clarified. Selenoprotein M (SELM) is an endoplasmic reticulum (ER)-resident thioredoxin-like enzyme. Downregulation of SELM is responsible for defective mitophagy and increased mitochondria-dependent apoptosis in MASLD via inhibition of the hepatocyte Parkin-AMPKα1-MFN2 signaling pathway [76]. Deletion of *Selm* exacerbates lipotoxicity, while *Selm* overexpression activates mitophagy to restore hepatocyte viability [76]. It seems that Parkin responds to AMPK-induced hepatocyte mitophagy in MASLD. As whole-body knockout lacks specificity for the liver and hepatocytes, the role of hepatocyte-specific *Slc7a11*, *Mst1,* and *Selm* deletion in hepatocyte mitophagy in MASLD may need to be explored. The direct role of hepatocyte AMPK should also be explored.

***PRDX6 signaling pathway***

In the antioxidant peroxiredoxin (PRDX) family, PRDX6 is the only member that translocates to impaired mitochondria. Increased PRDX6 expression in the mouse liver displayed great resistance to HFD-induced MASLD [80]. However, mitophagy levels were not fully assessed in this study. The protective effect of PRDX6 against hepatic lipid accumulation is mediated by enhancing mitophagy by suppressing Notch signaling in HepG2 cells [80]. Consistently, a more in-depth study revealed that PRDX6 and its binding protein, Myc-interacting zinc-finger protein 1 (Miz1), mediate the inhibition of Parkin-related mitophagy [81]. Moreover, impaired mitophagy caused hepatocyte inflammasome activation and stimulated macrophage TNF-α production in the MASH stage but not in the MASLD stage. TNF-α further contributes to Miz1 degradation, forming a vicious feedback loop [81]. Thus, impaired mitophagy may promote MASLD progression by inducing NLRP3 activation and pyroptosis in hepatocytes [82,83,84].

***P62 signaling pathway***

The autophagy adaptor protein p62 is known as downstream of ubiquitination. P62 targets ubiquitinated mitochondria to phagophores. In most cases, increased p62 levels indicate decreased consumption of the p62 protein and impaired autophagosome biogenesis (e.g., mitophagosomes), and vice versa. In comparison, blocking the p62-ZZ domain can reverse impaired mitophagy [85]. Notably, p62 mediates Parkin-independent mitophagy by recruiting Keap1 and Rbx1, two subunits of a cullin-RING ubiquitin E3 ligase complex [86]. The p62-Keap1-Rbx1 complex ubiquitinates mitochondria. However, the enhancement of enlarged mitochondria (termed megamitochondria) in MASLD hepatocytes suppresses Parkin-independent ubiquitination mediated by p62. In contrast, inhibiting the formation of megamitochondria can rescue impaired mitophagy and ameliorate liver injury [86]. This study establishes a link between p62-mediated mitophagy and MASLD progression.

Other p62-related signaling pathways also contribute to hepatocyte mitophagy. Olfactomedin 4 (OLFM4) is a glycoprotein whose expression is elevated in the livers of MASLD patients. OLFM4 interacts with p62 directly to promote hepatic mitophagy, whereas *Olfm4* knockout exacerbates MASLD by decreasing mitophagy via a p62-dependent mechanism [85]. Acyl-CoA lysocardiolipin acyltransferase-1 (ALCAT1) can catalyze pathological cardiolipin remodeling. In primary hepatocytes, ablation of *Alcat1* enhances autophagic flux, as indicated by elevated PINK1 and LC3-II and downregulated p62 levels [87]. The upregulation of hepatic ALCAT1 can inhibit PINK1-mediated mitophagy in hepatocytes, contributing to the pathogenesis of MASLD [87]. Mortality factor 4-like protein 1 (MORF4L1, also called MRG15) is stabilized on the OMM. MRG15 deacetylates the Tu translation elongation factor (TUFM), a critical regulator of virus-induced mitophagy, and accelerates its proteolytic degradation. Moreover, activation of the MRG15-TUFM pathway results in impaired hepatocyte mitophagy, increased oxidative stress, and activation of the NLRP3 inflammasome [88]. Overall, p62 impairs mitophagy in MASLD by interacting with the Keap1-Rbx1, OLFM4, ALCAT1, and MRG15-TUFM signaling pathways. However, the association between p62 signaling pathway-mediated mitophagy and MASH remains unclear and requires further elucidation.

***BNIP3 signaling pathway***

BNIP3 is a classic mitophagy receptor located on the OMM that mediates PINK1/Parkin-independent mitophagy. The livers of *Bnip3*-null mice display increased ROS levels, inflammation, and steatohepatitis, with an elevated proportion of abnormal mitochondria being observed simultaneously [89]. Endothelial nitric oxide synthase (eNOS) produces ROS at the expense of nitric oxide (NO) and maintains mitochondrial homeostasis in the liver. Low eNOS levels decrease BNIP3 expression in murine and human MASLD and MASH patients [90,91]. Furthermore, hepatocyte-specific *eNOS* deletion impairs hepatic BNIP3 expression, while hepatocyte *eNOS* overexpression partially rescues impaired mitophagy, dysfunctional mitochondria, hepatic steatosis, and inflammation [91].

Transcription factors, deacetylases, and E3 ubiquitin ligases can also regulate BNIP3 expression and mitophagy. Nuclear receptor subfamily 4 group A member 1 (NR4A1) is a transcription factor responsible for metabolic diseases. Upregulation of NR4A1 activates DNA-dependent protein kinase catalytic subunit (DNA-PKcs) to enhance p53 expression. Subsequently, the activation of p53 suppresses BNIP3 expression and causes mitophagy arrest. Moreover, genetic deletion of NR4A1 or DNA-PKcs reverses mitochondrial dysfunction and MASLD progression [92]. The deacetylase sirtuin 3 (SIRT3) is downregulated in response to high-fat stress. SIRT3 overexpression blocks hepatocyte mitochondrial apoptosis by activating the ERK-CREB signaling pathway and subsequent BNIP3-mediated mitophagy [93]. RNF31, an E3 ubiquitin ligase, is downregulated in HFD-fed mice and oleic-palmitic acid-treated hepatocytes. RNF31 promotes p53 degradation through ubiquitination. A reduction in p53 enhances the expression of BNIP3, thereby increasing mitophagy in hepatocytes. In vitro, the up-regulation of RNF31 in hepatocytes improves mitophagy and reduces lipid deposition and cell apoptosis. Overexpression of RNF31 ameliorates hepatic steatosis and improves liver function in mice with MASLD [94]. Thus, BNIP3-mediated mitophagy can be regulated by eNOS, NR4A1, DNA-PKcs-p53, RNF31-p53, and SIRT3.

***Other signaling pathways***

There are other potential targets related to hepatocyte mitophagy in MASLD. Paraoxonase-2 (PON2) is a membrane protein with an antioxidant effect. *Pon2* depletion results in the downregulation of Parkin, PINK1, and BNIP3L [95]. PON2 might help alleviate MASLD by promoting the sequestration of mitophagy cargo [95]. In contrast, regulated in development and DNA damage response-1 (REDD1) negatively regulates mitophagy. Loss of *Redd1* could protect against hepatic steatosis in HFD-fed mice, along with increased levels of autophagic and mitophagic markers, including Beclin, LC3-II, Parkin, and BNIP3L [96]. Although both PON2 and REDD1 are involved in regulating mitophagy in MASLD, the detailed underlying mechanisms require further analysis.

***The interplay between mitophagy and lipophagy in MASLD***

Lipid accumulation is one of the hallmarks of MASLD [5,6]. Mitophagy modulates the biogenesis of mitochondria and favors the β-oxidation of lipids in mitochondria [11,12]. In contrast, damaged mitophagy cannot maintain adequate healthy mitochondria, leading to liver steatosis and MASLD progression [8,96]. In addition to mitophagy, lipophagy also plays a pivotal role in MASLD pathogenesis. Lipophagy is a form of selective autophagy that participates in lipid homeostasis [97]. In the liver, hepatocytes are a crucial storehouse for free fatty acids. Free fatty acids transform into triglycerides for storage with cholesterol in lipid droplets (LDs) [98]. These LDs were generally thought to be metabolized merely by the process of lipolysis, until Singh et al. first confirmed the role of autophagy in modulating intracellular lipid storage [98]. Lipophagy targets LDs and generates free fatty acids. The released fatty acids then undergo β-oxidation in mitochondria [99]. Thus, mitophagy and lipophagy potentially interact. For example, the neutral lipase PNPLA5, which is localized to LDs, participates in both lipophagy and mitophagy [100]. Considering that abnormal accumulation of intracellular lipids impairs lipophagy [98], the interplay between impaired mitophagy and aberrant lipophagy may trap hepatocytes in a harmful cycle of increasing lipid retention and MASLD progression. However, the detailed signaling pathways involved in the interplay between mitophagy and lipophagy during MASLD pathogenesis require further investigation. A comprehensive understanding of the underlying mechanism, not limited to hepatocytes, will reveal more selective autophagy-related therapeutic targets for the treatment of MASLD.

Overall, the AMPK, PRDX6, p62, BNIP3, PON2, and REDD1 signaling pathways are involved in hepatocyte mitophagy in the context of MASLD. The interplay between mitophagy and lipophagy potentially influences MASLD progression. However, the hubs downstream of mitophagy receptors (SMRs or MMRs) are involved, and the crosstalk among these signaling pathways remains unclear. In addition, most studies are based on global knockout; therefore, hepatocyte-specific deletion of these hub genes is urgently needed for future in-depth studies.

### 3.2. Hepatocyte Mitophagy in Liver Fibrosis

Continuous insults induce hepatocyte injury and cell death, thereby initiating inflammation. Liver inflammation leads to the activation of HSCs, the major source of the fibrous scars in liver fibrosis [78,101]. Damaged mitophagy was demonstrated to be involved in carbon tetrachloride (CCl_4_)- or common bile duct ligation (CBDL)-induced liver fibrosis in rodents [102,103,104,105]. FK506 binding protein 51 (FKBP51) is a glucocorticoid receptor binding protein that is crucial for modulating metabolic function (Figure 3). *Fkbp51* knockout or an FKBP51 inhibitor has been shown to play a protective role (including reducing apoptosis) in CCl_4_-induced liver fibrosis by increasing Parkin expression [104]. In contrast, several studies have indicated hyperactivated mitophagy in the fibrotic liver [106,107,108]. Environmental aflatoxin B1 (AFB1) exposure can induce liver fibrosis. In response to AFB1 exposure, hepatocytes increase *Park2* transcription via p53 nuclear translocation and enhance mitochondrial recruitment of Parkin. The p53-Parkin axis promotes the intercellular crosstalk between hepatocytes and HSCs via AFB1-exposed hepatocyte-derived EVs (AFB1-EVs). Mitophagy-dependent AFB1-EVs are crucial for activated HSC-associated liver fibrogenesis [109]. The divergent role of mitophagy in liver fibrosis may be attributed to differences in the aminal models and cellular variance.

### 3.3. HSC Mitophagy in MASLD

**Figure 4:**
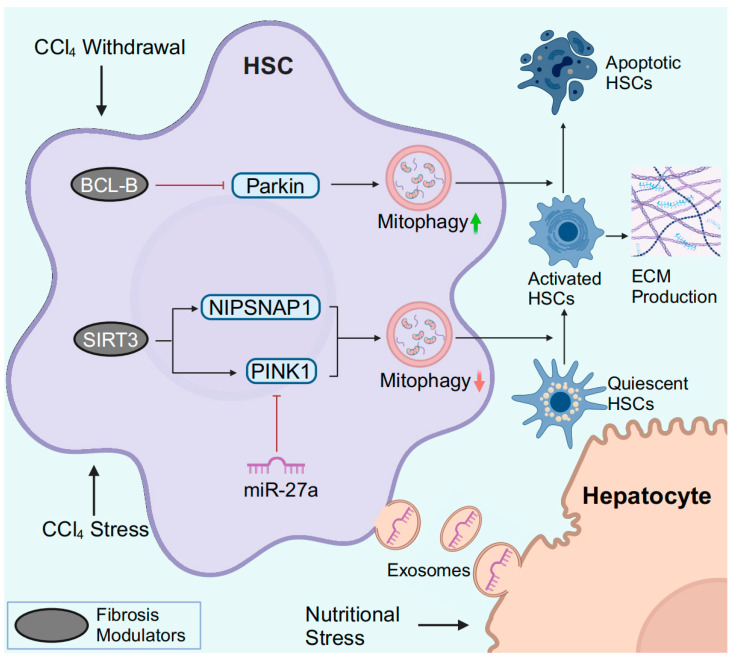
HSC mitophagy in MASLD and liver fibrosis. In response to CCl_4_, downregulated SIRT3 fails to specifically deacetylate PINK1 and NIPSNAP1, resulting in impaired mitophagy. During the process of CCl_4_ withdrawal, downregulation of BCL-B promotes HSC apoptosis by inducing Parkin-mediated mitophagy. Hepatocytes under nutritional stress release exosomal miR-27a to inhibit PINK1-mediated mitophagy in HSCs, leading to the activation of HSCs. Abbreviations: ECM, extracellular matrix. Created with BioRender.com (accessed on 30 March 2024).

In MASLD, the pro-inflammatory microenvironment facilitates quiescent HSC transdifferentiation into collagen-secreting activated HSCs (myofibroblasts). Activated HSCs are the major source of extracellular matrix (ECM) and promote liver inflammation by secreting cytokines [78]. MASLD may progress from a steatotic stage and/or active inflammatory early stages driven by metabolic syndrome to a fibrotic later phase. MASLD-related liver fibrosis involves mitophagy-related crosstalk between hepatocytes and HSCs (Figure 4). In response to lipotoxic injury, hepatocytes release exosomal miR-27a, which is preferentially absorbed by HSCs. In HSCs, miR-27a negatively regulates PINK1, which has a miR-27a binding site. Inhibition of PINK1 by lipotoxic hepatocyte-exosomal miR-27a impairs HSC mitophagy and promotes HSC activation and proliferation. Clinically, this crosstalk between hepatocytes and HSCs is correlated with the severity of liver fibrosis in MASLD patients [110]. Therefore, impaired HSC mitophagy may contribute to the progression of MASLD-induced fibrosis.

### 3.4. HSC Mitophagy in Liver Fibrosis

Chronic liver injury contributes to the activation of HSCs, the major source of excessive ECM in liver fibrosis. During the regression of liver fibrosis, activated HSCs switch to an inactivated phenotype (similar to but distinct from quiescent HSCs) or undergo apoptosis [101].

In vitro, Parkin-related mitophagy is induced and facilitates HSC activation in response to TGF-β stimulation [107]. Smad3 is a crucial mediator of TGF-β-induced mitophagy, but in-depth studies are lacking. PM2.5 refers to particulate matter (PM) with an aerodynamic diameter of less than 2.5 µm in the atmosphere. In PM2.5-induced liver fibrosis, PM2.5 activated ROS-mediated mitophagy and miR-411/Drp1-mediated mitophagy in HSCs [111,112]. In CCl_4_-induced murine liver fibrosis, decreased deacetylase SIRT3 levels are observed, while *Sirt3* knockout further exacerbates liver fibrosis [105]. Mitochondrial SIRT3 specifically deacetylates PINK1 and nonneuronal SNAP25-like protein 1 (NIPSNAP1) to improve impaired mitophagy and attenuate HSC activation and ECM production [105].

The clearance of activated HSCs by inducing HSC apoptosis is critical for reversing liver fibrosis (Figure 4). BCL-B is a member of the BCL-2 family and is known to regulate apoptosis. BCL-B suppresses HSC apoptosis by inhibiting the phosphorylation of Parkin and binding to phospho-Parkin [113]. During the recovery of CCl_4_-induced liver fibrosis, BCL-B is downregulated, and both mitophagy and apoptosis are induced in HSCs [113]. In addition, CCAAT/enhancer binding protein α (C/EBP-α) also induces apoptosis and mitophagy in activated HSCs [114]. However, the exact relationship between C/EBP-α-mediated apoptosis and mitophagy requires further in-depth analysis.

In summary, although some studies support that mitophagy is potentially induced during HSC activation, impaired mitophagy is more likely to occur in parallel with HSC activation, a pivotal event for liver fibrogenesis. In contrast, restored mitophagy facilitates the apoptosis of activated HSCs and reverses liver fibrosis. Thus, targeting HSC mitophagy may be a promising strategy for regulating the progression or blockage of liver fibrosis.

### 3.5. Macrophage Mitophagy in MASLD

Liver macrophages include different subpopulations, including liver-resident KCs and monocyte-derived macrophages (MoMs) [115]. KCs and freshly recruited MoMs exhibit protective functions in regulating inflammation and fibrogenesis [78,116]. Macrophages display different phenotypes in response to specific signals from their environment. According to a simplified classification, M1 macrophages are pro-inflammatory while M2 polarization refers to an anti-inflammatory phenotype [78]. On the one hand, macrophages protect the liver against pathogen invasion and facilitate tissue repair. On the other hand, overactivated macrophages exacerbate liver inflammation and fibrogenesis in MASLD.

**Figure 5:**
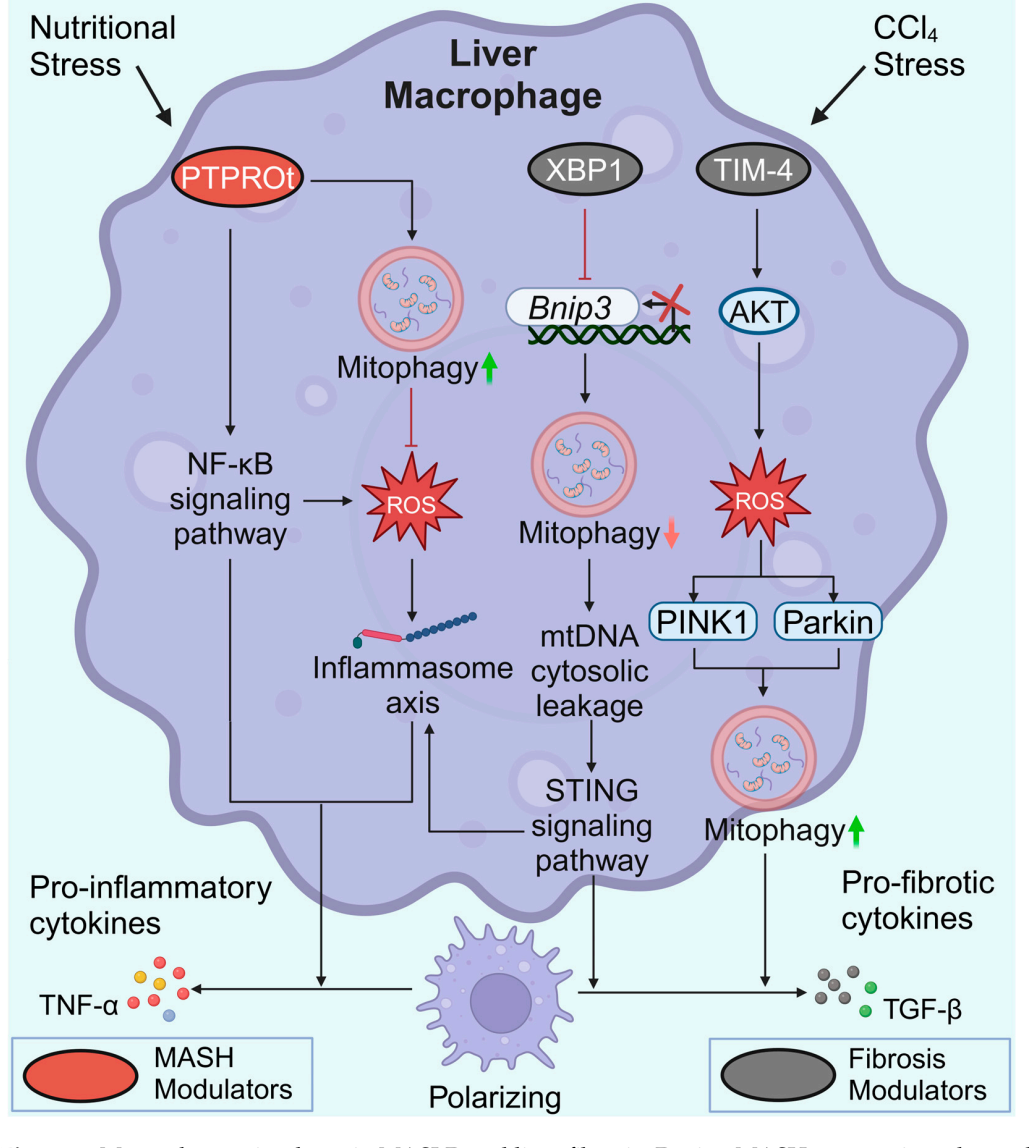
Macrophage mitophagy in MASLD and liver fibrosis. During MASH progression, elevated PTPROt expression exacerbates inflammation. However, PTPROt enhances mitophagy to partially restrict ROS production and inflammation. In liver fibrosis, XBP1 binds directly to the *Bnip3* promoter, suppressing the transcription of *Bnip3*. Impaired BNIP3-mediated mitophagy ultimately leads to increased release of pro-inflammatory and pro-fibrotic cytokines in macrophages. High TIM-4 levels contribute to increased levels of PINK1 and Parkin and the induction of TGF-β1. Created with BioRender.com (accessed on 29 May 2024).

The protein tyrosine phosphatase receptor type O truncated isoform (PTPROt) is an indispensable membrane protein for macrophages. Similar to the heterogeneity of macrophages, PTPROt may also play dual roles in liver fibrosis pathogenesis. In MASH mice, an increase in PTPROt in macrophages (KCs or MoMs) predominantly activated the NF-κB signaling pathway, further promoting ROS and the NLRP3/IL1β axis (Figure 5). In contrast, PTPROt can also activate mitophagy to partially restrict inflammation and ROS production, forming a negative feedback loop [117]. Considering the heterogeneity of macrophages, the role of macrophage mitophagy in MASLD and liver fibrosis is sophisticated. It is possible to speculate that hepatocyte lipoapoptosis is a crucial initial force of a pro-inflammatory environment in MASLD [116]. The pro-inflammatory environment activates M1 polarization of macrophages via suppression of mitophagy, thereby aggravating the pro-inflammatory environment. Sustained inflammation may lead to increased HSC activation and macrophages switching to a profibrotic phenotype, which subsequently promotes MASLD progression and fibrogenesis.

### 3.6. Macrophage Mitophagy in Liver Fibrosis

KCs and recruited MoMs are the primary cells involved in the inflammatory response. During the progression of liver fibrosis, the inflammatory response is a necessary process. Upon activation, KCs and recruited MoMs secrete pro-fibrotic cytokines (e.g., TGF-β1) and subsequently induce the differentiation of HSCs into myofibroblasts, which jointly promote liver fibrosis [78]. Additionally, macrophages can produce collagenases to degrade the ECM and support the regression of fibrosis [101].

T-cell immunoglobulin domain and mucin domain-4 (TIM-4) is selectively expressed on antigen-presenting cells. The activation of TIM-4 in KCs promotes fibrotic progression, whereas TIM-4 interference results in liver fibrosis resolution. Interfering with TIM-4 in KCs may suppress PINK1/Parkin-mediated mitophagy and decrease TGF-β1 levels by inhibiting AKT serine/threonine kinase 1 (Akt1)-mediated ROS production [118]. Conversely, elevated PINK1/Parkin-mediated mitophagy in KCs is required for TGF-β1 expression in liver fibrosis [118]. X-box binding protein 1 (XBP1) regulates the proinflammatory response in macrophages. In fibrotic liver macrophages, XBP1 binds directly to the *Bnip3* promoter to suppress *Bnip3* transcription. In contrast, *Xbp1* depletion restores BNIP3-mediated mitophagy to suppress the cGAS/STING/NLRP3 signaling pathway, thus protecting against liver fibrosis. However, the precise role of XBP1 and mitophagy in different macrophage subpopulations in regulating liver fibrosis remains to be explored [119]. Similar to the macrophage heterogeneity, macrophage mitophagy has different effects on liver fibrosis. Increased macrophage mitophagy in MASLD can partially limit inflammation and ROS production [117], whereas elevated PINK1/Parkin-mediated mitophagy is required for TGF-β1 expression and fibrogenesis [118]. Due to limited research and the heterogeneity of macrophages, it remains unknown which type of mitochondrial autophagy in macrophages is more influential in the process of liver fibrosis.

### 3.7. LSEC Mitophagy in MASLD

LSECs form the vascular wall of the hepatic sinusoids. LSECs are characterized by a lack of an organized basement membrane and the presence of transcellular pores [120]. Under healthy conditions, LSECs help maintain hepatic homeostasis. During the progression of MASLD, LSECs become capillarized (termed capillarization) and exhibit an abnormal angiocrine signaling, partially leading to inflammation, lipid deposition, and liver fibrosis [121]. LSEC capillarization can precede MASLD and lead to the progression and perpetuation of MASH [120]. Recently, a single-cell RNA sequencing analysis identified a damaged LSEC population in methionine-choline deficiency (MCD)-induced MASH mice. A cluster of C-Kit^+^ (KIT proto-oncogene receptor tyrosine kinase)-LSECs was confirmed to restore PINK1-related mitophagy and alleviate MASH progression [122]. A study in EC transplantation revealed that mitochondria transfer enhances the engraftment ability of ECs by triggering PINK1/Parkin-related mitophagy after internalization [123]. Due to limited research on LSEC mitophagy in MASLD, further investigations of LSEC mitophagy are needed.

### 3.8. LSEC Mitophagy in Liver Fibrosis

In response to sustained liver injury, LSECs exhibit a switch from pro-regenerative to pro-fibrotic phenotype, with LSECs becoming capillarized. Pro-fibrotic LSECs contribute to dysregulated sinusoidal homeostasis, aberrant liver healing, and fibrosis progression [121]. As few studies focus on LSEC mitophagy in liver fibrosis, we speculate that LSEC mitophagy might be involved in the phenotypic switch of LSECs. Targeting LSEC mitophagy might be a potential modality for reversing LSEC capillarization. Due to the crucial role of LSECs in modulating cellular crosstalk via angiocrine signaling [121], LSEC mitophagy might also influence hepatocyte regeneration, HSC transformation, and macrophage infiltration and polarization.

Overall, most of the mitophagy-related basic experimental investigations in MASLD and liver fibrosis have focused primarily on hepatocytes, HSCs, and macrophages. However, the role of mitophagy in other liver cells, including LSECs, cholangiocytes, neutrophils, T cells, and B cells, is limited or unknown. Much more attention should be given to these cells, especially LSECs, which are crucial regulators of cellular interplay within the liver microenvironment. In addition, mitophagy-related crosstalk between liver cells requires further exploration.

## 4. Mitophagy as a Target for the Treatment of MASLD and Liver Fibrosis

Currently, only resmetirom is now approved along with diet and exercise for the clinical treatment of adults with MASH-related liver fibrosis (F2–F3) but not for those with liver cirrhosis (F4). Thus, exploring potential strategies or compounds for treating liver cirrhosis is urgently needed. Considering the significant role of mitophagy in MASLD and liver fibrosis, we summarized mitophagy-related therapeutic strategies or compounds for MASLD and liver fibrosis (Table 1). These strategies range from non-pharmacological physical exercise and caloric restriction to pharmacological Chinese herbal formulas, antioxidant agents, diabetic medications, natural products, and other small molecules or peptides. Almost all strategies do not specifically target mitophagy. We described their impact on mitophagy as fully as possible to provide ideas for the development of new drugs. In addition, gut microbiota composition/alteration-induced mitophagy and mitophagy-related clinical trials are discussed.

***Physical exercise and caloric restriction***

Physical exercise and caloric restriction have long been recognized as valuable non-pharmacological strategies to counteract MASLD by improving hepatic mitophagy [124,125,126,142]. Both voluntary physical activity and endurance training (ET) positively affect the mitochondria of MASH rats, but only the ET group positively modulates the expression of PINK1 and Parkin [126]. Caloric restriction appears to restore impaired mitophagy, while a weight-loss diet plus physical exercise has a more significant effect on restoring mitophagy and ameliorating MASLD symptoms in male mice [124]. Swimming exercise also partially restores the number of mitophagosomes, increases the expression of Parkin, decreases p62 levels, and improves mitochondrial quality in MASLD zebrafish livers [142]. Very few FDA-approved pharmacological methods specifically target MASLD. Resmetirom is approved for treating MASH-related liver fibrosis in conjunction with diet and exercise. Physical exercise and dietary intervention seem to provide alternative approaches to rescue aberrant mitophagy and mitochondria in MASLD.

***Antioxidant***

Antioxidants are man-made or natural compounds that inhibit oxidation. AntiOxCIN4 is a mitochondrion-targeted antioxidant based on caffeic acid. Supplementation with AntiOxCIN4 has been demonstrated to mitigate hepatic steatosis in Western diet-fed mice with MASLD via a series of mechanisms, including potentially restoring mitophagy [127]. Mitoquinone (MitoQ) mesylate is a well-known mitochondrion-targeted antioxidant with high bioavailability. In rats with CBDL, MitoQ facilitates the clearance of damaged mitochondria by increasing Parkin translocation to mitochondria [103]. In addition, MitoQ inhibits the activation of HSCs by promoting PINK1/Parkin-mediated mitophagy [128]. Interestingly, elevations in PINK1 and Parkin levels are observed in mice fed an HFD, whereas PINK1 in mouse liver tissue can be reversed by the ROS scavenger N-acetyl cysteine (NAC) [129]. These results demonstrate the great potential of mitochondria-specific antioxidants for treating MASLD and liver fibrosis by improving mitophagy.

***Traditional Chinese Medicine/Classical Herbal Formula***

Traditional Chinese medicine (TCM) is a complete medical system that has been used to diagnose, treat, and prevent diseases for more than 2000 years. In classical TCM herbal formulas, each herb has a different purpose or role to help the body synergistically achieve harmonious harmony. The Shugan Xiaozhi formula, a classical TCM herbal formula, alleviates MASH by enhancing BNIP3/BNIP3L-mediated mitophagy in HFD-induced murine models [130]. With abundant antioxidant ingredients, the Ger-Gen-Chyn-Lian-Tang formula decreases lipotoxicity in db/db mice with MASLD by promoting mitophagy via Parkin-dependent and Parkin-independent signaling pathways [131]. The Yang-Gan-Jiang-Mei formula promotes PINK1/parkin-mediated mitophagy and subsequently perturbs NLRP3 inflammasome activation, suppressing hepatocyte inflammation in rats fed a HFD [132]. In brief, dysfunctional mitochondria may be degraded or rehabilitated by specific phytochemical component-mediated mitophagy. However, it remains challenging to clarify the comprehensive mechanisms underlying traditional herbal formulas because of their complicated components. Specific effective elements need to be identified, and their precise connection to mitophagy is expected to be explored.

***Diabetic medications***

Since MASLD is strongly associated with type 2 diabetes, diabetic medications are also important for the management of patients with MASLD. Metformin is an initial treatment strategy for type 2 diabetes. Metformin has an optimal effect on MASLD in obese rats when combined with caloric restriction compared with either therapy alone. This therapeutic effect is associated with an increase in the mitophagy marker BNIP3 [133]. Both exenatide and liraglutide are glucagon-like peptide-1 (GLP-1) receptor agonists. Male MASLD mice treated with exenatide exhibited an increased number of autophagosomes, elevated expression of LC3A/B-II/I, Beclin-1, Parkin, and BNIP3L, and suppression of the NLRP3 and IL-1β proteins [143]. Similarly, liraglutide slows the progression of MASH by inhibiting NLRP3 inflammasome-induced hepatocyte pyroptosis by enhancing PINK1-related mitophagy [83]. We can gain insights from these published studies, e.g., that the therapeutic effects of diabetic drugs on MASH might be partially derived from improved mitophagy.

***Others***

Many other pharmacological approaches exert a protective effect on MASLD and liver fibrosis. Natural products or compounds, including corilagin, quercetin, corn peptides, and cyanidin-3-O-glucoside (C3G), are abundant in plants. Corilagin mitigated HFD-mediated mitophagy blockade, as evidenced by increased Parkin levels and enhanced colocalization of Parkin and mitochondria [134]. Quercetin, corn peptides, and C3G improve mitochondrial dysfunction and lipid accumulation via PINK1/Parkin-mediated mitochondrial autophagy [135,136,137,138]. Notably, quercetin, a natural compound, mitigates MASLD by promoting frataxin-mediated and AMPK-mediated mitochondrial autophagy [135,136]. In addition to these pharmacological ingredients being abundant in plants, particular attention has also been given to small molecules or peptides. Melatonin is an indoleamine synthesized by the pineal gland. Melatonin supplementation blocks the NR4A1/DNA-PKcs/p53 signaling pathway, resulting in the inhibition of Drp1-mediated mitochondrial fission, the recovery of BNIP3-related mitophagy, and subsequent improvement of mitochondrial and liver function in mice with MASLD [92]. Impaired PINK1/Parkin-mediated mitophagy is also rescued by melatonin in CCl_4_-treated rats [102]. Moreover, melatonin suppresses ROS-induced mitophagy to partially alleviate murine liver fibrosis induced by PM2.5 [108]. These results indicate that melatonin has a hepatoprotective effect by either rescuing impaired mitophagy or inhibiting hyperactivated mitophagy. S-acetyl-glutathione (SAG) is an S-acyl prodrug capable of replenishing endogenous glutathione. SAG administration protects against liver fibrosis by restoring PINK1 and Parkin expression [139]. Pyrvinium pamoate, an anti-adipogenic compound, also attenuates the progression of MASH by exerting a pro-mitophagic effect [140]. As an agonist of adiponectin receptors, JT003 degrades the ECM but fails to halt the production of harmful elastin-derived peptides. Thus, JT003 combined with the V14 peptide (an elastin-derived peptide inhibitor) has a synergistic effect on mitigating MASLD and MASH-related liver fibrosis [141]. This synergistic effect may be attributed to increased mitophagy and mitochondrial biogenesis induced through the AMPK signaling pathway.

***Gut Microbiota intervention***

In MASLD and liver fibrosis, disturbance of the gut microbiota has been observed and is promoted by damaged intestinal barriers [144]. For example, proteobacteria are consistently enriched in stages of steatosis and steatohepatitis [145]. Sustained perturbation of the gut-liver crosstalk facilitates liver inflammation and disease progression [144]. Recent studies have validated the potential link between gut microbiota and liver mitophagy in xenobiotics-induced liver disease. Upon cadmium-induced liver injury, melatonin supplementation alters the gut microbiota profile, leading to an increased production of gut-derived LPS. The involvement of gut microbiota mediates melatonin efficacy in mitigating mitophagy, ER stress, and liver inflammation, and improving the oxidation of fatty acids [146]. Melatonin also mitigates liver mitophagy and inflammation induced by ochratoxin A, a widespread contaminant, via reversing gut microbiota dysbiosis and restoring intestinal barrier function [147]. Gut microbiota disturbance also mediates battery wastewater-induced hepatotoxicity via a gut-liver axis and the involvement of mitophagy and liver apoptosis [148]. 

Fecal microbiota transplantation (FMT) is a promising therapeutic modality for treating human diseases. Gut microbiota intervention has been shown to have mitophagy-related therapeutic effects in liver disease. Oral fecal gavage enriched in *Lachnospiraceae* and butyrate mitigates acute liver injury induced by acetaminophen. Notably, the butyrate induces mitophagy and Nrf2 antioxidant responses, thereby alleviating acute liver injury [149]. The natural compound puerarin has been demonstrated to enhance mitophagy in HFD-induced obese mice, which is correlated with improved gut health [150]. However, investigations of mitophagy-related gut microbiota intervention in MASLD and liver fibrosis are still limited. Further analysis, especially the causal role of the gut microbiota in mitophagy during MASLD and liver fibrosis is needed.

***Mitophagy-related Clinical trials***

Both non-pharmacological and pharmacological activation of mitophagy have been shown to protect against MASLD and liver fibrosis in experimental animals. However, there is only one mitophagy-related clinical trial investigating the treatment of MASLD or liver fibrosis. This randomized double-blinded placebo-controlled clinical trial (NCT05526144) aims to test whether low-dose thyroid hormone administration could improve biopsy-proven MASH in veterans, in which BNIP3 is the secondary outcome measurement. Therefore, additional translational studies are required to further validate these novel mitophagy modulators for the treatment of MASLD and liver fibrosis.

## 5. Prospects and Conclusions

Mitophagy is an evolutionarily conserved cellular process that removes and recycles impaired mitochondria. Thus, mitophagy fine-tunes mitochondrial function and number and maintains cellular metabolic homeostasis. In most cases, damaged cell mitophagy leads to hepatocyte injury, the activation of HSCs, and the polarization of pro-inflammatory macrophages, thereby facilitating the progression of MASLD and liver fibrosis. In contrast, restoring mitophagy damage reduces hepatocyte injury and death, potentially induces activated HSC apoptosis, and negatively modulates macrophage inflammation and secretion of pro-fibrotic cytokines. LSEC mitophagy may play a dual role but requires further investigations. Mitophagy may also be harmful for MASLD and liver fibrosis. Under certain circumstances, activated mitophagy leads to hepatocyte injury (e.g., hepatocyte ferroptosis) and the activation of HSCs and pro-fibrotic macrophages. These controversial effects of mitophagy could be attributed to differences in experimental conditions, liver cell heterogeneity, or mitophagy overactivation. Adequate suppression of overactivated mitophagy might partially improve MASLD and liver fibrosis. Further analysis to clarify the role of mitophagy in different liver cells and the balance between impaired and hyperactivated mitophagy is of great importance.

Additionally, several issues remain to be solved. (1) Although many modulators and signaling pathways have been demonstrated to participate in liver cell mitophagy in MASLD and liver fibrosis, the detailed relationship between mitophagy regulators and downstream mitophagy has not been elucidated. In vitro experiments do not represent the actual role of the corresponding liver cells in vivo. Whole-body genetic modification cannot exclude effects from other organs or cells. Therefore, more investigations based on cell-specific genetic modifications are urgently needed for future in-depth studies. (2) Most studies have focused on vacuolation, steatosis, oxidative stress, inflammatory injury, and fibrosis in the liver. However, the liver is a central organ that modulates metabolism in the body. Multiple ‘hits’ underlie the mechanism of onset of MASLD. The link between mitophagy and other features of MASLD, including insulin sensitivity, glucose homeostasis, and malfunctional adipose tissues, requires further analysis. (3) The role of mitophagy in other non-parenchymal liver cells in MASLD and liver fibrosis remains unknown. Only a few studies have described mitophagy-related crosstalk between liver cells. More in-depth mechanistic studies are needed to clarify the landscape and novel therapeutic targets of liver mitophagy in MASLD and liver fibrosis. Single-cell-based multi-omics combined with deep learning-based artificial intelligence may help. (4) Most mitophagy-related strategies or compounds for treating MASLD and liver fibrosis are based on experimental animals and cell models. More translational studies are anticipated to further validate these newly identified mitophagy regulators for the clinical treatment of MASLD and liver fibrosis.

In conclusion, impaired mitophagy in hepatocytes, HSCs, and macrophages significantly participates in the pathogenesis of MASLD and liver fibrosis. Mitophagy-related therapeutic strategies or compounds might be translational for the clinical treatment of MASLD and liver fibrosis.

## Figures and Tables

**Table 1:** Mitophagy-related therapeutic strategies or compounds for MASLD and liver fibrosis.

	Strategies or Compounds	Disease	Model	Changes in Mitophagy	References
Physical exercise and caloric restriction	WR plus CR	MASLD	Mice	Increased levels of BNIP3, PINK1, and Parkin	[124]
	WR	MASLD	Mice	Induced upward trends in transcription and expression of *Bnip3*	[125]
	Endurable WR	MASH	Rats	Increased expression of PINK1 and Parkin	[126]
Antioxidant	AntiOxCIN_4_	MASLD	Mice	Induced an upward trend in Parkin levels	[127]
	MitoQ	Liver fibrosis	Rats, mice	Increased translocation of Parkin to mitochondria in rats; promoted PINK1/Parkin-mediated mitophagy in mouse HSCs	[103,128]
	NAC	MASLD	Mice	Inhibited highly activated expression of PINK1	[129]
Traditional Chinese medicine/classical herbal formula	SG formula	MASH	Mice	Increased BNIP3/BNIP3L-related mitophagy	[130]
	GGCLT formula	MASLD	Mice, db/db mice	Increased Parkin-dependent/independent mitophagy	[131]
	YGJM formula	MASH	Rats	Promoted PINK1/Parkin-mediated mitophagy	[132]
Diabetic medications	Metformin plus CR	MASLD	OLETF rats	Increased BNIP3 levels	[133]
Others	Corilagin	MASLD	Mice	Increased Parkin levels; enhanced colocalization of Parkin and mitochondria	[134]
	Quercetin	MASLD	Mice	Increased PINK1/Parkin-mediated mitophagy	[135,136]
	Corn peptides	MASLD	Rats	Activated PINK1/Parkin-mediated mitophagy	[137]
	C3G	MASLD	Mice	Increased PINK1-mediated mitophagy	[138]
	Melatonin	MASLD	Mice	Restored BNIP3-related mitophagy in hepatocytes	[92]
		Liver fibrosis	Mice	Suppressed activated PINK1/Parkin-mediated mitophagy in hepatocytes treated with particulate matter	[108]
		Liver fibrosis	Rats	Restored PINK1 and Parkin levels	[102]
	SAG	Liver fibrosis	Mice	Restored PINK1 and Parkin levels	[139]
	PP	MASH	Rats	Restored PINK1/Parkin-mediated mitophagy	[140]
	JT003 plus V14 peptide	MASLD	Mice	Increased BNIP3 levels; expression of mitophagy-related genes (e.g., *Park2*, *Bnip3l*); and colocalization of lysosomes with mitochondria	[141]

Abbreviations: WR, wheel running; CR, caloric restriction; NAC, N-acetyl cysteine; MitoQ, mitoquinone; SG formula, Shugan Xiaozhi formula; GGCLT formula, Ger-Gen-Chyn-Lian-Tang formula; YGJM formula, Yang-Gan-Jiang-Mei formula; C3G, cyanidin-3-O-glucoside; SAG, S-acetyl-glutathione; PP, pyrvinium pamoate; JT003, an AdipoR1/2 dual agonist; V14 peptide, an inhibitor of elastin-derived peptides; Mice, C57BL/6 mice; Rats, Sprague Dawley rats; db/db mice, type 2 diabetic mice; OLETF rats: Otsuka Long-Evans Tokushima Fatty rats.
